# Acceptance procedure for the linear accelerator component of the 1.5 T MRI‐linac

**DOI:** 10.1002/acm2.13068

**Published:** 2021-07-17

**Authors:** Simon J. Woodings, J. H. Wilfred de Vries, Jan M. G. Kok, Sara L. Hackett, Bram van Asselen, Johanna J. Bluemink, Helena M. van Zijp, Theo L. van Soest, David A. Roberts, Jan J. W. Lagendijk, Bas W. Raaymakers, Jochem W. H. Wolthaus

**Affiliations:** ^1^ Department of Radiotherapy University Medical Center Utrecht Utrecht The Netherlands; ^2^ Elekta AB Stockholm Sweden

**Keywords:** acceptance tests, dosimetry, magnetic field, MRI‐linac, Unity

## Abstract

**Purpose:**

To develop and implement an acceptance procedure for the new Elekta Unity 1.5 T MRI‐linac.

**Methods:**

Tests were adopted and, where necessary adapted, from AAPM TG106 and TG142, IEC 60976 and NCS 9 and NCS 22 guidelines. Adaptations were necessary because of the atypical maximum field size (57.4 × 22 cm), FFF beam, the non‐rotating collimator, the absence of a light field, the presence of the 1.5 T magnetic field, restricted access to equipment within the bore, fixed vertical and lateral table position, and the need for MR image to MV treatment alignment. The performance specifications were set for stereotactic body radiotherapy (SBRT).

**Results:**

The new procedure was performed similarly to that of a conventional kilovoltage x‐ray (kV) image guided radiation therapy (IGRT) linac. Results were acquired for the first Unity system.

**Conclusions:**

A comprehensive set of tests was developed, described and implemented for the MRI‐linac. The MRI‐linac met safety requirements for patients and operators. The system delivered radiation very accurately with, for example a gantry rotation locus of isocenter of radius 0.38 mm and an average MLC absolute positional error of 0.29 mm, consistent with use for SBRT. Specifications for clinical introduction were met.

## 1 INTRODUCTION

**Fig. 1:**
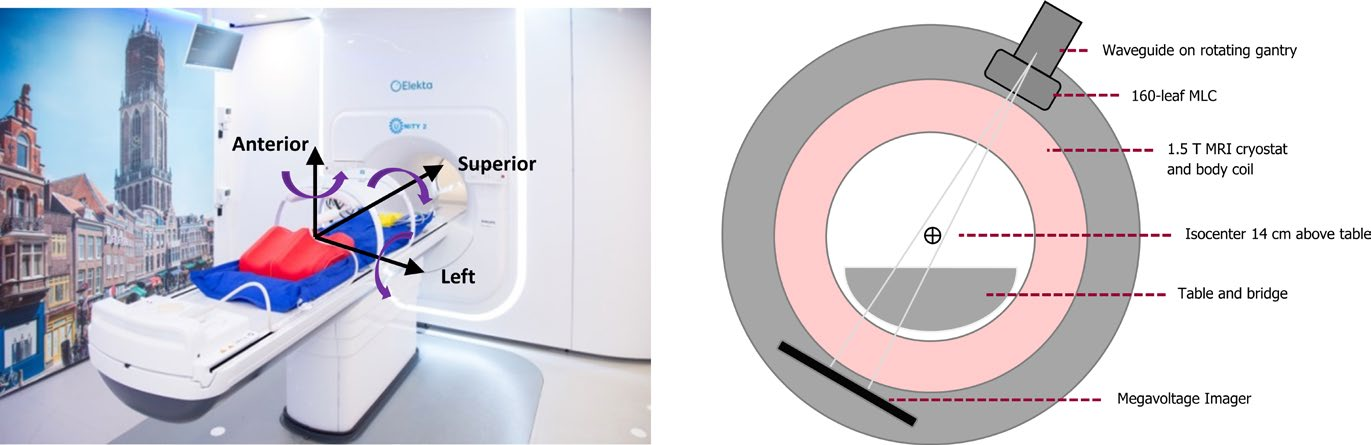
Left; Image of the first clinical Elekta Unity MRI‐Linac at UMCU, including head‐first supine patient coordinates, and right; schematic showing basic linac and system features

Elekta AB (Stockholm, Sweden), Philips (Best, The Netherlands) and University Medical Center Utrecht (UMCU) have developed a linear accelerator (linac) with integrated 1.5 T magnetic resonance imaging (MRI) (Fig. 1). This combination facilitates simultaneous irradiation and high‐precision image guidance with soft‐tissue contrast.^1^ Elekta Unity (MRI‐linac) is the clinical implementation of the prototype machine described by Raaymakers et al.^2^ The system is in clinical use.^3^, ^4^

To safely and effectively deliver radiation beams from this machine to a patient, it was necessary to perform an acceptance testing and commissioning process. The fundamentals of this process have been well established in the Medical Physics and Radiotherapy communities and are documented by, for example, the IEC,^5^ AAPM,^6^, ^7^ IPEM,^8^, ^9^ and NCS.^10^, ^11^ However, due to the special characteristics of this machine, new methods, equipment, and tests had to be developed.

### 1.1 MRI‐linac special characteristics

The Elekta Unity has a 1.5 T magnetic field which points out of the bore (Fig. 1). The presence of the magnetic field means that all equipment, including for example water phantom drive motors, must be MR‐compatible.

Dose measurements and dose distributions are affected by the B_0_ field. The Lorentz force acts on charged particles, pulling them in a direction orthogonal to both their vector motion and the magnetic field. This has a small effect inside and outside the radiation beam, but is clearly noticeable where there is an absence of electronic equilibrium—at interfaces and beam edges.^12^ This effect must be taken into account in assessment of beam symmetry, beam edges, beam alignment, and alignment of beam limiting devices. Furthermore, the electron return effect (ERE) must be characterized.^13^ Finally, charged particles can also stream along magnetic field lines, which must be considered during radiation safety tests (eg dose to patient)^14^ and during clinical implementation.

The 70 cm diameter wide‐bore system is large enough for most patients but is a limit on equipment size. The isocenter is not easily accessible from outside the bore and therefore most equipment is setup externally using lasers or templates on the table, and then transferred with precise table motion to isocenter, similarly to other radiotherapy systems with bores (eg Halcyon (Varian Medical Systems, Palo Alto California, USA), Tomotherapy (Accuray, Madison Wisconsin, USA), and MRIdian (Viewray, Oakwood Village Ohio, USA)). There is no light field, therefore the gantry‐mounted megavoltage imager (MVI) is used for equipment position verification and for the daily morning QA check (Fig. 1).

The radiation beam passes through the liquid helium‐filled, multi‐layered metal cryostat, and the MRI body coil. These have been designed to be as homogeneous as possible but the transmission varies with gantry angle and must be characterized for the TPS. The cryostat and coil are a source of scattered radiation. This requires additional characterization measurements and affects the beam model and radiation safety.^13^ The level of liquid helium is stable, but can change after interventions such as magnet ramp‐up or ramp‐down. To minimize the effect of any change in the liquid helium level on clinical dose delivery, Elekta recommend that the beam is calibrated with a beam from gantry 90 degrees.

The Philips Marlin MRI (version 2017‐04‐10 at time of writing) has been designed with a cylindrical beam portal in the windings of the magnet so that the radiation beam can pass in between. This gap allows a maximum field size in the superior‐inferior (*y*) direction of 22 cm at the isocentric depth, which is a limitation on clinical use at this moment. Due to the extended SAD a field size of up to 57.4 cm can be achieved in the lateral direction. This is important to optimally treat peripheral targets. Wider water phantoms have also been designed especially to accommodate QA tests for this field size.

The system delivers single‐energy 7 MV flattening filter free (FFF) step‐and‐shoot intensity modulated radiation therapy (IMRT) beams from a standing wave waveguide mounted on a solid ring gantry around the MRI with a source axis distance (SAD) of 143.5 cm (Fig. 1). The system does not use steering coils and therefore there is much less chance of the beam steering being incorrect, or changing over time, which reduces the number of QA tests on linac stability. The internal monitor unit ion chambers are sealed.

The table is fixed with comfort mattress 13.0 cm below, and solid surface 14.0 cm below, isocenter. The table supports only longitudinal motion for initial patient setup and phantom setup. A treatment plan must be optimized based on images acquired of the patient in their current treatment position. Table accuracy, axis, angle, and flex are not critical for clinical use, but they are important for setup of any phantoms which cannot be MR imaged. As the table is fixed, the radiation isocenter will typically not be within the target region and the MRI‐linac will routinely deliver small off‐axis radiation fields. Thus greater attention to these is required during beam characterization.^13^ The linac gantry cannot tilt and the table cannot rotate so all MRI‐linac treatments are co‐planar. All beams have central axis perpendicular to the magnetic field.

The MLC is based on the Elekta Agility model with 80 leaf pairs with rounded leaf ends, each with projected width of approximately 0.72 cm at isocenter. The collimator does not rotate so it cannot be used to define a mechanical or radiation isocenter, and therefore a new method is needed. MLC leaves move always in the superior‐inferior (*y*) directions. The MRI‐linac MLC has the additional capability to park opposing leaves underneath the primary collimator. The MLC is fully inter‐digitating and can thus create island fields for simultaneous irradiation of multiple target regions. Minimum opposing leaf separation is set to 0.5 cm on central axis at isocenter in the initial configuration, equivalent to 0.1 cm between opposing leaves in the leaf bank. Full‐attenuation diaphragms move in the orthogonal (*x*) direction.

The MRI system is used for all patient imaging. Acceptance testing and commissioning of the MRI has previously been described{Tijssen, 2019 #61}.

Coordinate system transformation (alignment) between the MR imaging and MV delivery systems is critical and therefore must be tested.

Radiation delivery and MR imaging can be performed simultaneously without reducing image quality, and without affecting the radiation beam.^2^, ^15^

The magnet is linked across the plane of linac gantry rotation with a superconducting wire in a conduit. The conduit also allows the helium pressure to equalize across the whole system. The wire and conduit are centered within the beam at a gantry angle of 13°. Direct irradiation of this wire is excluded by the system. Thus there are limitations on beams delivered from approximately 13°, that depend upon field size and gantry angle and are incorporated into the treatment planning system (TPS).

### 1.2 Aims

The aims of this work were to:

1. Create an acceptance testing procedure with reference to existing protocols;
2. Describe modified and new tests and equipment;
3. Demonstrate that the procedure could be performed, and to provide results of the tests from the first Unity MRI‐linac.

## 2 MATERIALS AND METHODS

### 2.1 Phantoms and detectors

There are special considerations for phantoms and detectors within a high‐magnetic field environment. Phantoms must contain minimal ferromagnetic materials, which precludes their standard electric motors, power supplies, batteries, electronics, and drive arms.^16^ Air cavities must be eliminated to avoid perturbations to detector readings.^17^, ^18^, ^19^

Several phantoms were used for acceptance tests. Ion chamber scanning in water was performed with an Elekta‐Philips prototype MR‐compatible MP3‐style scanning water phantom, whose design was based on an earlier prototype.^16^ Reference dosimetry was established with a PTW prototype MR‐compatible MP1 (1D) water phantom (PTW, Freiburg, Germany) with a manual depth drive. Routine dosimetry was performed with a RW3 phantom consisting of multiple 30 x 30 x 1 cm^3^ slabs and a prototype RW3 slab with a Farmer chamber cavity that was sealed with water around the chamber to prevent any air layer affecting the detector reading.^17^ Other RW3 slabs, with chamber cavities filled with ultrasonic gel, were used for various point dose measurements. A polymethylmethacrylate (PMMA) buildup cap with diameter 3.2 cm (with water‐filled cavity), was used with a Farmer chamber for comparison of radiation beams from different gantry angles.

Elekta provided several phantoms for specific tasks. The Elekta MV Geometry Phantom was used to measure the isocenter coordinates on the MV imager (MVI). The Elekta MR‐MV phantom, with multiple ceramic ball bearings mounted in a CuSO4‐filled framework, was used to measure the coordinate transformation between the MRI and the MV coordinate systems. The Elekta Las Vegas phantom and the Standard Imaging (Middleton, USA) QC3 phantom were used to measure MVI image quality. The Elekta Pixel Tool, a precision‐milled 2D brass plate, was used to measure the MVI pixel size and panel tilt.

The readings in air‐filled ion chambers are dependent on the relative orientations of radiation beam, magnetic field and chamber axis, and on chamber size, beam energy, and field strength. These differences are due to the varying average path length of the ionising track of a secondary charged particle and the inflow of electrons into the chamber.^20^ Additional correction factors are required for absolute dosimetry.^21^, ^22^, ^23^, ^24^, ^25^ Relative dosimetry measurements in a scanning water phantom within the expected range of conditions can still be made with <0.3% error due to chamber orientation.^16^

PTW30013 and FC65‐G (IBA‐Dosimetry, Schwarzenbruck, Germany) Farmer‐type waterproof chambers were used with a collecting voltage of −250 V for absolute dosimetry. Waterproof chambers were chosen to enable measurements in water (or with water‐filled phantom cavities) to prevent dosimetric artifacts from air layers around the detectors.^17^ PTW Semiflex3D detectors were used for medium and large‐field relative dosimetry. The effective point of measurement (EPoM) of the Semiflex3D within a 1.5 T magnetic field was determined by comparing the percentage depth dose build up curve to that measured with a PTW60019 microDiamond. The resultant EPoM was very close to the −0.5 mm value used by UMCU and the −0.3 *r*_cyl_ = −0.7 mm recommended by O’Brien et al.^26^ The PTW60019 microDiamond detector was used with an EPoM of +1.0 mm for small‐field measurements and for assessing beam penumbra.^27^ A PTW Tandem electrometer was used with the Elekta‐Philips phantom for collecting profile data. PTW Unidos E and Unidos Webline electrometers were used for point data collection.

A PTW31010 Semiflex detector was used as a monitor (reference) ion chamber. The chamber was mounted on an arm above the water surface and inside the corner of a 5 x 5 cm^2^ field. It was later observed that for best results with the scanning water phantom the reference chamber should be placed well above the water surface (private communication, PTW).

2D array measurements were made with a PTW StarCheck maxi MR and a Sun Nuclear IC Profiler MR (Melbourne, USA).

The megavoltage imager (MVI) plays an important role in the alignment of the various measurement equipment. The Perkin Elmer (Santa Clara, USA) amorphous silicon detector is rigidly mounted on the gantry ring, aligned opposite to the beam at a source‐detector distance (SDD) of 2658 mm. It has 1024 x 1024 pixels over an area of 410 x 410 mm^2^. The physical pixel size and positions are rescaled to the isocenter distance. The MVI is located at a position on the gantry ring outside the MR cryostat where the magnetic field strength is close to 0^28^ (see Fig. 1). Thus the MVI data are free of magnetic field induced artifacts and remains an effective surrogate of the photon fluence.

Film sandwiched between copper sheets (thus capturing all generated electrons) was also used to measure a surrogate of photon fluence that was independent of Lorentz force^29^. Absolute position on a coronal film was acquired, if necessary, by simultaneous imaging with the MVI and then registering the film image to the MVI image and position.

### 2.2 Acceptance tests

**Fig. 2:**
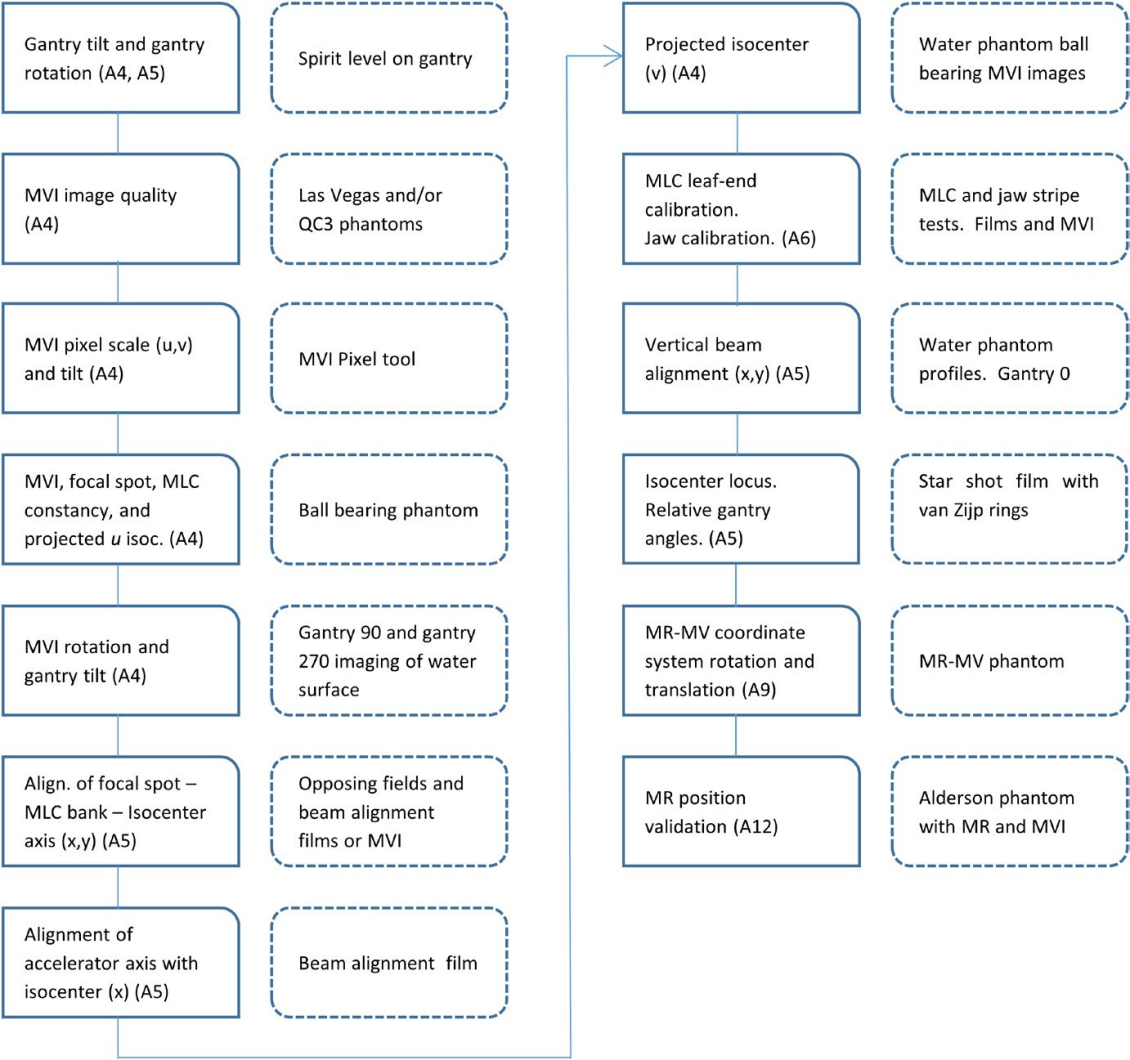
Schematic of independent system alignment checks for the linac component of the MRI‐linac

The acceptance tests are presented in categories. The acceptance tests are listed in Table 1. A logical and efficient order in which tests should be done, based on dependencies and efficiency, is shown in Fig. 2.

**Table 1:** Linear accelerator acceptance tests and specifications. External references for tests and specifications are shown in brackets

Section	Description	Phantom	Specification
A1	**Safety**		
	Inhibit systems		
A2	**Radiation shielding**		
	Scattered radiation to the patient	Mini‐phantom	ave < 0.1% of in‐field dose (IEC 60601‐2‐1)
	Bunker protection		<1.0 mSv/year
A3	**Coordinate systems and data integrity**		
	Radiation beam and beam shaping (IEC61217)		
	MVI (IEC61217)		
	MRI (DICOM)		
A4	**MVI**		
	Panel rigidity	Ball bearing	<0.3 mm
	Rotational alignment	Water phantom	<0.2^o^
	Pixel scale and isocenter	Elekta pixel tool, MV alignment phantom	
	Image quality	QC3, Las Vegas	
A5	**Mechanical and dosimetric alignment of gantry, focal spot and beam**		
	Gantry tilt	Spirit level	<0.2^o^
	Gantry rotation and readout	Spirit level and spoke film	<0.2^o^
	Beam alignment	Film, water phantom	<1 mm and <0.2^o^
	Isocenter locus	Film	<0.5 mm radius
A6	**Mechanical and dosimetric alignment of MLC and jaws**		
	MLC stripe test	Film, MVI	<0.5 mm (TG 142)
	MLC transmission	Film, water phantom	<0.5% from baseline/TPS (TG142)
	Jaw stripe test	Film, MVI	<1 mm (TG 142)
	Gantry angle dependency	Film, MVI	<0.5 mm (TG 142)
A7	**Table**		
	Orthogonality and movement	MVI	<1° and <2 mm (TG 142)
A8	**Laser — Elekta indicative sagittal laser**	Ball bearing	<1 mm (TG 142)
A9	**MR to MV alignment**	Elekta MR‐MV alignment phantom	<0.3^o^ (Elekta)
A10	**Dosimetric system**		
	Output stability — short and long term	RW3	<2% (TG 142)
	2^nd^ linac monitor chamber	RW3	<2% (TG 142)
	Linearity	RW3	<2% (TG 142)
	Dose rate dependency	RW3	<2% (TG 142)
	Output gantry angle and cryostat dependency	Mini‐phantom	<1% from baseline (TG 142)
	Profile constancy	StarCheckMaxi	<1% (TG 142)
	Profile gantry angle dependency	ICprofiler	<1% (TG 142)
	Internal monitoring and beam cutoff	ICprofiler	3%
	Reference dosimetry	Water phantom	<1% (NCS 18)
A11	**Beam performance during MR imaging**	Water phantom	<2%
A12	**End‐to‐end test**	Alderson phantom	γ (5%/2mm), 10% threshold, >90% pixels passed

### 2.3 A1 Safety

Standard safety inhibit and interlock tests included the Ferroguard ferrous materials detection system (Metrasens Inc, Lisle Illinois, USA), last‐person‐ring‐out system, door interrupt, radiation warning lights and audio, camera functionality, function keypad interrupt and terminate buttons and lights, machine room door inhibit on gantry motion, table drive, and emergency on button. Functionality and calibration were checked for lasers, heating ventilation, and air conditioning (HVAC) system condensation, SF6 gas level and bottle weight, mains power supply lights, cellar door interlock, magnet‐on light, and flood alarm.

The linac contains a second, independent, multi‐channel ion chamber for beam checking. Functionality of this second dose channel, as well as difference between the two channels (dose difference interrupt) and beam energy (uniformity and dose‐per‐pulse interrupts) were checked during the Elekta Device Acceptance Tests (DAT). Changing the gun grid voltage can change the beam doserate, output, quality, and profile. With assistance from Elekta, the change in dose distribution for a beam just within the linac internal interrupt tolerances was quantified. The maximum differences in output and beam quality were measured with an ion chamber in RW3. Maximum differences in profile (off axis dose) were measured with an IC profiler.

### 2.4 A2 Radiation shielding

A limit of 1.0 mSv/y was applied outside the treatment bunker, consistent with national (The Netherlands) and international guidelines (IAEA SRS 47 ^30^). Primary and secondary shielding walls, and the maze and door were assessed using standard methods.^30^ Neutron dose measurements were performed.^30^ Doses within the plane of the patient, and doses around the head of the linac were measured as per IEC 60601.^31^

### 2.5 A3 Coordinate systems

The radiation delivery system (linac) uses the IEC61217 coordinate system^32^ with the origin at isocenter. The imaging system uses DICOM coordinates. The megavoltage imager (MVI) rotates with the gantry, and therefore its coordinate system also rotates with respect to the patient. Its origin is in one corner of the panel (gantry 0 degrees, head first supine patient superior, right direction). The watertank has a coordinate system which can be modified in its software settings. Here it was set for consistency with IEC 61217. The coordinate systems are compared in Table 2.

**Table 2:** Coordinate systems used in the Elekta Unity system and in this article, compared to a head first supine (HFS) patient (see Fig. 1)

HFS Patient	IEC 61217 fixed	DICOM	MVI G0^o^	MVI G90^o^
Left	+x	+x_DICOM_	+u	
Superior	+y	+z_DICOM_	‐v	‐v
Anterior	+z	‐y_DICOM_		‐u

Asymmetric phantoms and plans were used to check coordinates and orientations of sub‐systems. The consistency of the whole Unity system was confirmed in the end‐to‐end test (section A12).

### 2.6 A4 MVI tests — alignment and isocenter

The Megavoltage Imager (MVI), formerly electronic portal image device (EPID), is not intended for patient imaging. It is the fundamental device used for QA and verifying equipment position inside the bore of the MRI‐linac. It also plays a significant role in the independent system alignment checks shown in Fig. 2.

As it is such a fundamental device for QA, the MVI panel rigidity was tested with a 5 x 5 cm^2^ MLC‐only beam delivered from 12 gantry angles (each 30 degrees apart). A ball bearing was placed within foam blocks close to isocenter. The ball bearing location and the beam edges were measured in each image. The ball bearing projected *u* position varied sinusoidally with gantry angle due to its offset from the isocenter. This effect was fitted and corrected. Theoretically any residual variation in the imaged ball bearing location (*u,v*) would be due to either (a) MVI panel shift (b) focal spot shift or (c) gantry ellipticity. The uncertainty of the measurements was ~ 0.5 pixels = 0.1 mm. Any variation in the field edge positions would be due to the same effects and/or MLC shift.

**Fig. 3:**
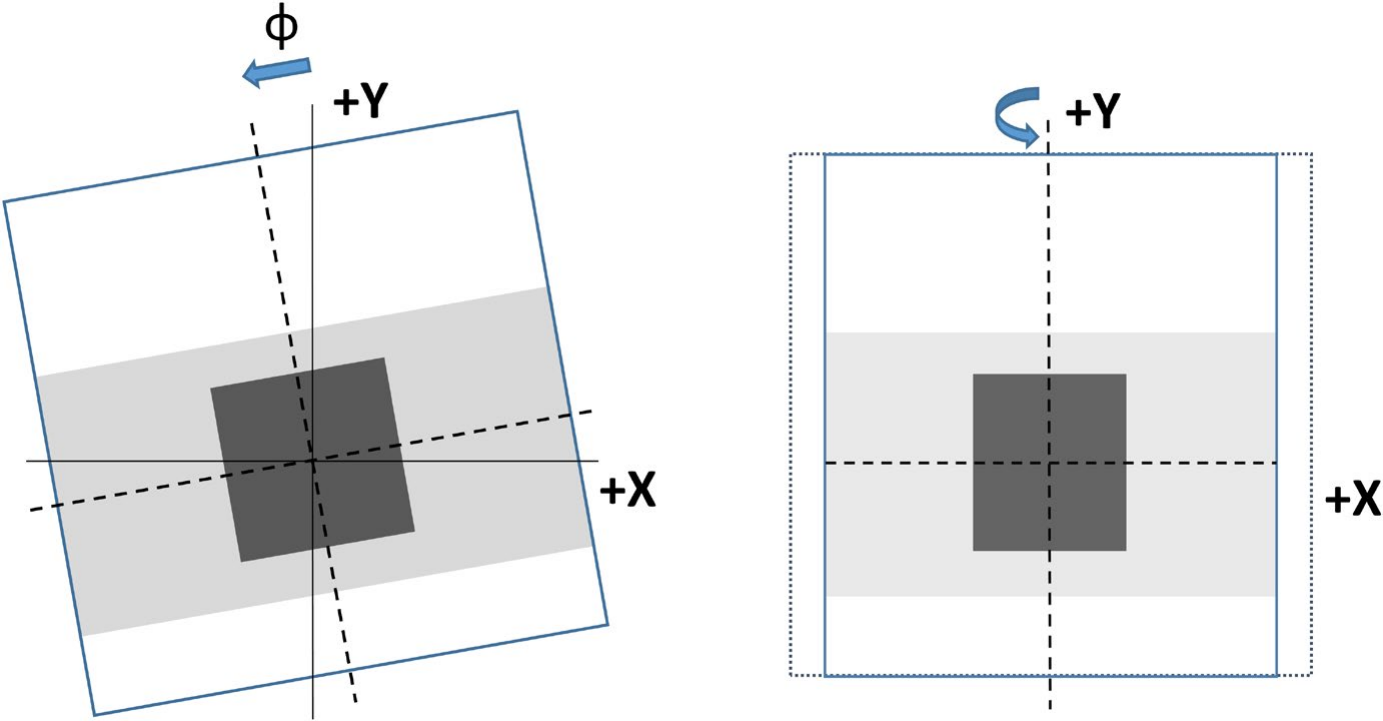
Schematic of MVI panel (a) rotation φ around z axis and (b) tilt around y axis. Nominal beam shown is from gantry 0°

MVI panel rotational alignment with the IEC 61217 coordinate system (see Fig. 3) was tested with the MP1 water phantom with the water surface nominally at isocenter. The water surface was imaged from gantry 90° and 270°. The angle (α) of the MV image pixel columns relative to the water surface at 90° (α_90_) and 270° (α_270_) were measured (but are not shown in the figure). Gantry tilt (θ_gantry_) and MVI panel rotation (φ_MVI_, shown in Fig. 3) were calculated from the angles using equations 1 and 2.

(1) $$\varphi_{\mathrm{MVI}}=\left(\alpha_{90},+,\alpha_{270}\right)/2$$

(2) $$\theta_{\mathrm{gantry}}=\left(\alpha_{90},‐,\alpha_{270}\right)/2$$

MVI image pixel scale was measured in both (*u* and *v*) directions using the Elekta Pixel Tool with known dimensions, levelled at isocenter. Any difference between the measured dimensions in the two directions would be due to panel tilt (Fig. 3). MVI images were acquired from both gantry 0° and 180° and the results averaged, which cancelled any small variation due to setup.

Isocenter coordinates (*u*_isoc_,*v*_isoc_) projected on the MVI panel were determined using the Elekta device acceptance test (DAT) method. The isocenter, the central point in the vertical *x‐z* plane defined by gantry rotation of the focal spot, was accurately determined in the x and z directions (*u*_isoc_) by imaging the Elekta geometry phantom ball bearing from multiple (12) directions. In the y direction, the projected position of the center of the beam limiting devices was used. A V‐shape in the back of each jaw was imaged for several jaw positions using special Elekta service beams. These images were then processed with Elekta software to determine the *v*_isoc_ coordinate. An approximate check of the *v* position was performed by imaging a ball bearing at multiple positions within the scanning water phantom.

MVI image quality was measured with the Las Vegas phantom and with the QC3 phantom, each at isocenter. The Las Vegas phantom was assessed visually and as per the Elekta DAT. The QC3 phantom was analyzed to determine the modulation transfer function (MTF) expressed equivalent to line pairs per mm, as per a previous published result from the MRI‐linac prototype.^33^ The predefined phantom analysis tools in Theraview (Cablon Medical, Best, the Netherlands) were used.

### 2.7 A5 Mechanical and dosimetric alignment of gantry, focal spot and beam

Gantry tilt (rotation around the + x axis) was determined with a spirit level, and from the MVI panel rotation measurement (see subsection A4 and Fig. 2). Spirit level measurements were performed against the gantry frame (not a reference surface) for a number of different gantry rotations.

Gantry rotation and readout (around the + y axis) were measured with a spirit level on the reference surface of the Elekta beam generation system (waveguide). Measurements were made at 270 and 240 degrees, where the reference surface is accessible from the machine room.

An MLC‐only spoke film was irradiated with beams from 12 different gantry angles delivered to a Gafchromic EBT3 film (Ashland, New Jersey USA) mounted in a transverse plane through isocenter.^29^ The relative angles of the beams were then analysed, as well as the radius of the locus of isocenter.

Constancy of alignment of the focal spot and the MLC with gantry angle was tested as part of the MVI panel test.

Waterphantom *x* and *y* profiles were collected at depths of 1.3, 5.0, and 10.0 cm and analysed to check whether the beam was vertical. Field edges defined by the point of inflection of the penumbra were analysed and compared.

Precise overlap of the gantry 0° and gantry 180° beams was checked with an opposing fields film.

**Fig. 4:**
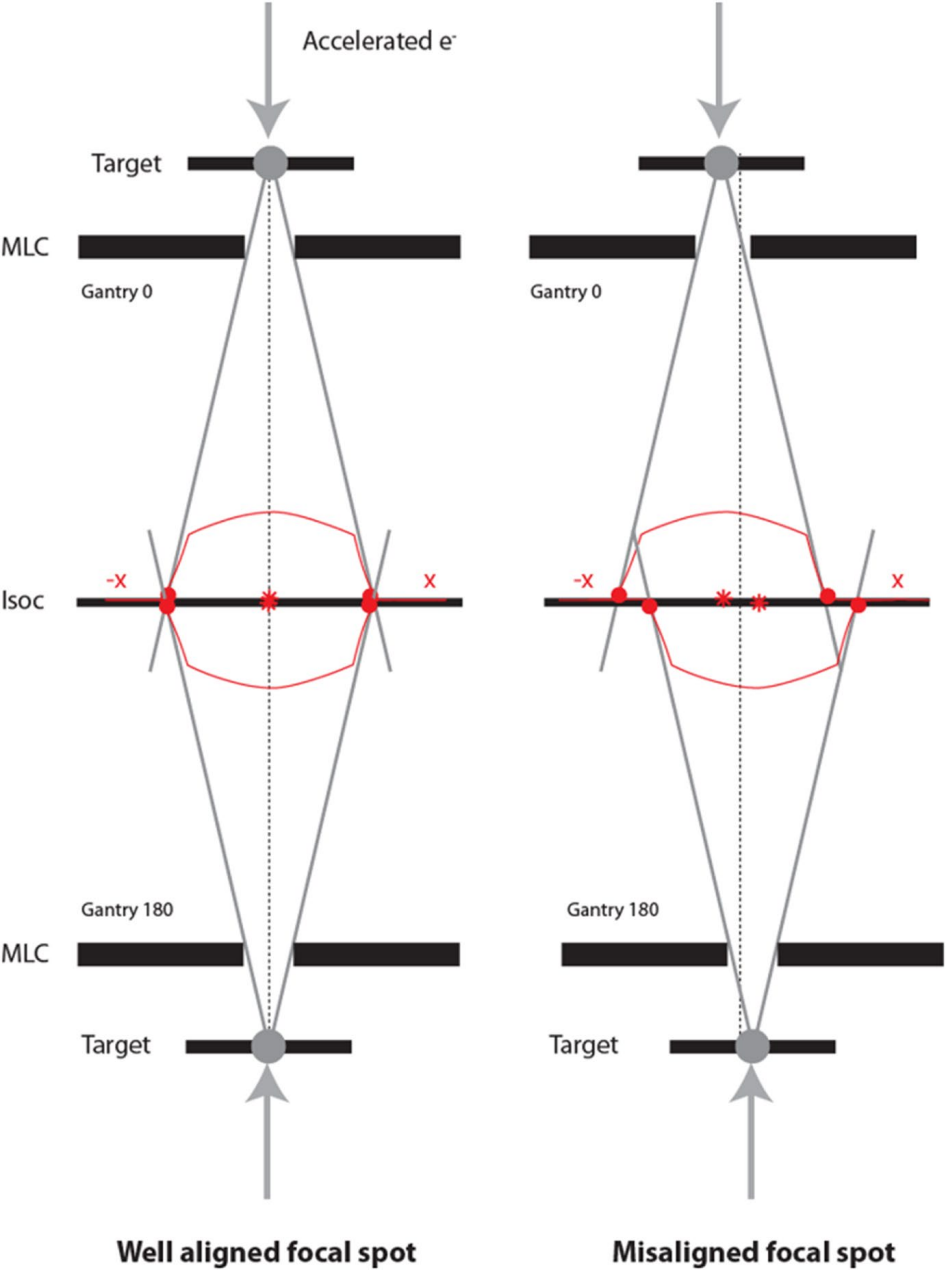
Schematic (front view) of the effect of a focal spot misalignment by using two opposed beams. Film check can be performed for jaw edges, MLC sides, and for FFF peak position (beam angle)

A check of the beam alignment based on relative FFF peak position was performed with gantry 0 and gantry 180 degree MLC‐only half‐beams (Fig. 4). A film in the coronal plane through isocenter was irradiated and x‐profiles analyzed. The profiles were also co‐registered based on the beam edges to check the position of the MLC bank (sides of the MLC).

A check was also performed by Elekta to verify that the maximum field size in the *y* direction fitted between the two halves of the MRI primary magnet.

### 2.8 A6 Mechanical and dosimetric alignment of MLC and jaws

The operation and calibration of the Elekta Unity multileaf collimator (MLC) has been previously described and the transmission quantified.^13^ The MLC and jaws were calibrated using the in‐built Elekta workflows. The MLC and jaw calibrations were independently checked with UMCU tests (Fig. 2).

A stripe test with 1 mm leaf overlaps every 20 mm was measured with a film (between copper sheets) and the MVI over the range—70 < y < +70 mm for the central 56 leaf pairs. In‐house software was used to assess the profile minimum due to relative leaf overlap, and the center of each overlap region (absolute positioning error).^34^ The high‐resolution EBT3 film data measured at isocenter was registered to the MVI image that was simultaneously acquired, so that the MVI‐based coordinate system could be transferred to the film. A visual assessment of constancy was performed at gantry 0° for all 80 leaf pairs. Average offsets and standard deviations were assessed and reported for each leaf pair and for each stripe.

The jaws were verified with fluence film measurements of 50 mm wide stripes over the range –200 to +150 mm. The positions were evaluated using in‐house software that found the point of inflection of each edge. Maximum and average offsets and standard deviations were reported.

The stripe test was repeated at the cardinal gantry angles for the central 30 leaf pairs using the MVI.

### 2.9 A7 Table

Table orthogonality and precise movement are not critical for the MRI‐linac, where each treatment is adapted based on the new position of the patient for each fraction. They are required for precise phantom setup.

Orthogonality and movement with and without load (90 kg) were assessed. For three different indexed couch positions the Elekta geometry phantom was mounted, the couch moved to the predefined position and the phantom imaged. The position of the ball bearing was assessed using the in‐built Elekta software. Residual translational offsets in the cardinal directions were recorded. Additional checks of orthogonality of the table and the table movement to the plane of the gantry were made using a set square, couch index bar, and MVI images.

### 2.10 A8 Lasers

Lasers are not required for the MRI‐linac as the treatment plan will be adapted to the patient position for each fraction. The installed system did include an indicative sagittal laser for approximate setup.

### 2.11 A9 MR to MV alignment

The rotational and translational offsets between the MRI coordinate system and the linac (MV) coordinate system were measured using the in‐built Elekta workflow and the Elekta MR to MV phantom (Fig. 2). Using the pre‐defined 3D MR imaging protocol and the predefined x‐ray beam sequence resulting in 31 MVI images, the MR to MV offset was calculated using the in‐built Elekta software. The rotational alignment had to be within 0.3 degrees. The translational offset was entered into the Unity software environment so that the Monaco TPS could accurately place the MV isocenter within the MR dataset prior to adaptation of treatment plans.

### 2.12 A10 Dosimetric system

The linac was calibrated consistent with the Netherlands Code of Practice NCS18^35^ with additional correction factors for the influence of the magnetic field^25^:

(3) $$D_{w,Q}^{B}=c_{B}k_{B,M,Q}M_{Q}^{B}N_{D,w,Q_{0}}k_{Q,Q_{0}}$$

where $D_{w,Q}^{B}$ is the absorbed dose to water, $c_{B}$ is the ratio of dose with magnetic field over dose without magnetic field, $k_{B,M,Q}$ is the ratio of ionization reading without magnetic field over reading with magnetic field, $M_{Q}^{B}$ is the ionization chamber reading with magnetic field corrected for influence quantities, and $N_{D,w,Q_{0}}k_{Q,Q_{0}}$ are the chamber calibration coefficient and beam quality correction factors.

The linac was calibrated to deliver 70.1 cGy per 100 MU at isocentre (143.5 cm) at 10 cm depth (SSD 133.5 cm) for a 10 x 10 cm^2^ beam delivered from gantry 90°. This was equivalent to a *D*_max_ of 100 cGy per 100 MU at *d*_max_ = 1.3 cm under these reference conditions. The dose rate was automatically set to 425 MU/min with a pulse repetition frequency (PRF) of 275 Hz and a gun duty cycle of 71% (percentage of radio frequency pulses that are accompanied by gun pulses). The measurements were performed with both PTW30013 and IBA FC65‐G Farmer‐type waterproof chambers. The chambers were placed horizontally in an in‐house built RW3 phantom, perpendicular to the radiation beam and anti‐parallel to the magnetic field. The RW3 phantom factor was derived from comparisons of measurements in the RW3 phantom and the MP1 water phantom.

The values of linac monitor chamber dose channels 1 and 2 were calibrated for 200 MU. Short term constancy was tested with 10 exposures in a row. Long term constancy was initially tested over a period of two weeks, with results here reported for a period of four months. Linearity with dose was tested over the range 2 – 1000 MU. Linearity with dose rate was tested for 54, 297 and 418 MU/min by varying the gun grid duty cycle between 10%, 50%, and 71% (the standard operating value for this linac).

Profiles were tested for different gantry angles and for 5 MU and 100 MU using an ICprofiler on the Elekta rotating platform during installation. This system allowed measurements to be acquired without the bridge or table affecting the beam. The crossline symmetry was expected to show greater variation due to (i) the Lorentz force shift of the profile (ie that the dose profile should be slightly asymmetric) and (ii) variable transmission through the cryostat with gantry angle.

### 2.13 A11 Beam performance during MR imaging

Output and planar relative dose distributions were measured with and without simultaneous MR imaging to test constancy. A beam was delivered from gantry 0° to a film at isocenter at depth 10 cm. A typical imaging sequence was used: 3D Turbo gradient field echo (TFE) with field of view 500 × 500 × 150 mm^3^, resolution 1.3 × 1.3 × 4 mm^3^, bandwidth 508.6 Hz/pixel, repetition time (TR) 6.5 ms, echo time (TE) 3.6 ms, flip angle (FA) 13°, T1 contrast enhancement, 102 TFE shots, TFE factor 100, number of signal averages (NSA) 4. Radiofrequency (RF) power optimization and field (f0) determination were switched off.

This test was repeated for the cardinal gantry angles with a 18 × 7 cm^2^ beam imaged with the MVI.

Ion chamber measurements were performed without scanning, and during a number of different MRI scan sequences; 2D T2 turbo spin echo (TSE), T1 TSE and a diffusion weighted image (DWI).

### 2.14 A12 End to end test

An end‐to‐end test requires the whole system, not only the linac component and was therefore not strictly within the scope of this article. Nevertheless, it is noted for the interest of the reader that an end‐to‐end test was performed using film in an Alderson anthropomorphic phantom (last step in Fig. 2). The film position was defined by markers placed within the phantom. The lymph node plan was adapted based on the treatment position of the phantom. Dose magnitude and position were assessed.

## 3 RESULTS

With only one photon energy, no flattened fields, no wedge and no electron beams, and with a beam line that cannot be user‐adjusted, there were less acceptance tests than on a kV‐IGRT linac. Some of the tests were new, but they were generally not more complex. When performed on the first Elekta Unity clinical system, the results of all tests were acceptable for clinical use. Test results for each category are presented below.

### 3.1 Safety

The safety tests were passed.

Changing the gun grid voltage to deliberately induce a fault condition changed the beam doserate, output, quality, and profile causing an inhibit. Within the deliverable range, the maximum change in TPR_20,10_ was 0.4%, in output was 1.8%, and in off‐axis dose was 1.3% (maximum point difference within the central 80% of field size of a 30 × 22 cm^2^ beam). Thus the internal inhibits were effective in preventing the beam from deviating substantially from its calibrated state.

### 3.2 Radiation shielding

All doses were within the national and international regulated limits. Within the patient plane, the highest measured leakage dose was 0.02% of the in‐field dose, within the 0.2% (max) and 0.1% (average) specification.^31^ Neutron readings were at the background level.

### 3.3 Coordinate systems

Coordinate systems and orientations were checked for each sub‐system and throughout the Unity system and found to be consistent at all times with those listed in Table 2.

### 3.4 MVI

The position (rigidity) of the MV imaging panel had a standard deviation of 0.06 mm *u* and 0.03 mm *v*. Field edges were also constant to within 0.07 mm. Assuming no synchronization between focal spot shift, gantry ellipticity, MLC shift and panel shift, the panel rigidity is better than 1σ = 0.06 mm.

The angles of the MV image pixel columns relative to the water surface were each measured to be α_90_ = α_270_ = 0.5/400 pixels = +0.072° and from equations 1 and 2 gantry tilt (θ_gantry_) was calculated to be 0.0 ± 0.1° and MVI panel rotation (φ_MVI_) was calculated to be 0.07 ± 0.1°. The MVI rotational misalignment could conceivably be taken into account in the evaluation of other acceptance tests. However, here it was established that installation of the panel was accurate (within 0.1 degrees) and the rotation was then considered negligible for other tests.

The MVI pixel scale was determined to be 0.2519 mm/ pixel, with no difference in the *u* and *v* directions and therefore no panel tilt. MVI panel isocenter coordinates (*u*_isoc_,*v*_isoc_) were determined to be (512.01, 651.64) (with origin pixel (1,1)). For use on the MVI display (with a (0,0) origin) the isocenter coordinates are (511.01,650.64). Independent UMCU measurements determined (510.5 ± 1.2, 652 ± 12), where the *u* uncertainty was 2σ (standard deviation with coverage factor k = 2) and the *v* uncertainty was estimated assuming a water phantom setup uncertainty of 0.1°. The Elekta and UMCU results were consistent. The Elekta values were applied for all further tests.

The modulation transfer function (MTF) was equivalent to 0.3 line pairs per mm, consistent with the results from conventional linear accelerators (0.4 lp/mm), taking into account the extended SAD, detector distance, focal spot size, and magnification factor. This was consistent with previously published results from the MRI‐linac prototype.^33^

### 3.5 Mechanical and dosimetric alignment of gantry, focal spot and beam

Gantry rotation and readout were assessed. The direct angle measurements at 270° and 240° agreed within 0.1 ± 0.1°. The relative gantry angles measured from the spoke film had standard deviation 0.13° and maximum deviation 0.25° all ±0.35°.

Gantry tilt was determined by spirit level and by laterally imaging a water surface. Both methods were consistent that the gantry tilt was 0.0 ± 0.1°.

**Fig. 5:**
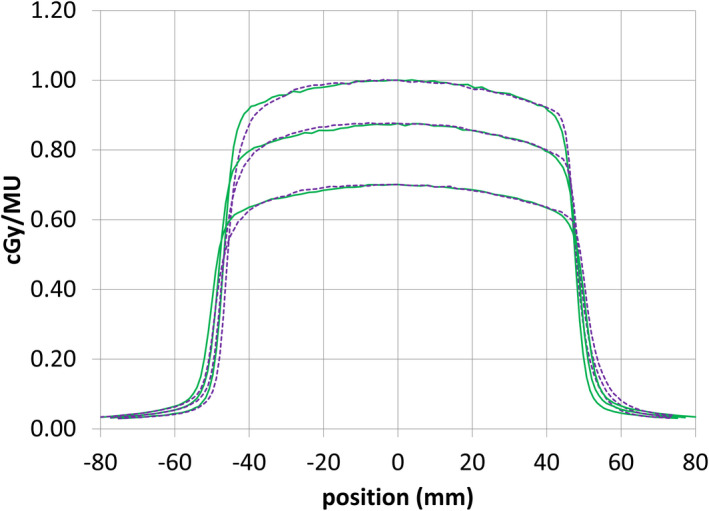
Inline (green, solid) and crossline (purple, dashed) profiles at 1.3, 5, and 10 cm depth for a 10 × 10 cm^2^ beam

Waterphantom *x* and *y* profiles were analysed to check whether the beam was vertical based upon analysis of the beam edges (Fig. 5). The Lorentz force was expected to create a constant + *x* offset at each depth. The center of the crossline scans (based on field edges) shifted by −0.2 mm and the center of the inline scans by −0.4 mm, which implied that the “vertical” beam was travelling at an angle of 0.13 ± 0.2° around the y axis and −0.26 ± 0.2° around the x axis. The test was dependent upon the reproducibility of the gantry angle (< 0.1°), the water phantom setup (< 0.1°) and the small range of depths measured (1.3 – 10 cm) (<0.1°). The combined uncertainty in the determined angle was < 0.2°. The clinical impact of an error of 0.3° would be a worst‐case 1 mm error in the beam location at a distance of 200 mm from isocenter.

From the MVI rigidity measurements at different gantry angles, the average MLC field center was (u,v) = (0.01, 0.09) mm from the Elekta defined isocenter position. The standard deviation around this average position was σ = (0.07, 0.06) mm. This implied that the MLC origin was well matched to the isocenter, and that the position was valid over all gantry angles.

The film to check beam alignment showed that the focal spot was centrally aligned between the MLC sides, with an acceptable offset of 0.3 mm.

The gantry 0° beam FFF peak crossed the patient plane (coronal plane through isocenter) at x = −1.6 mm from isocenter corresponding to a beam angle of 0.06 ± 0.1 degrees, which was considered acceptable. Uncertainty in the measurement was due to noise, potential systematic effects in the film and film scanner, and the gentle slope of the MRI‐linac FFF beam. Adjustment of the direction of the MRI‐linac beam can only be achieved with physical movement of the waveguide, and should not be needed after installation.

The opposing fields film showed that the beam centers from gantry 0° and gantry 180° were aligned to within 0.63 mm crossline and −0.13 mm inline.

The field sizes of the opposing fields were also measured. The maximum difference was 0.6 mm, which was within the tolerance of 2 mm (1 mm per field edge).

**Fig. 6:**
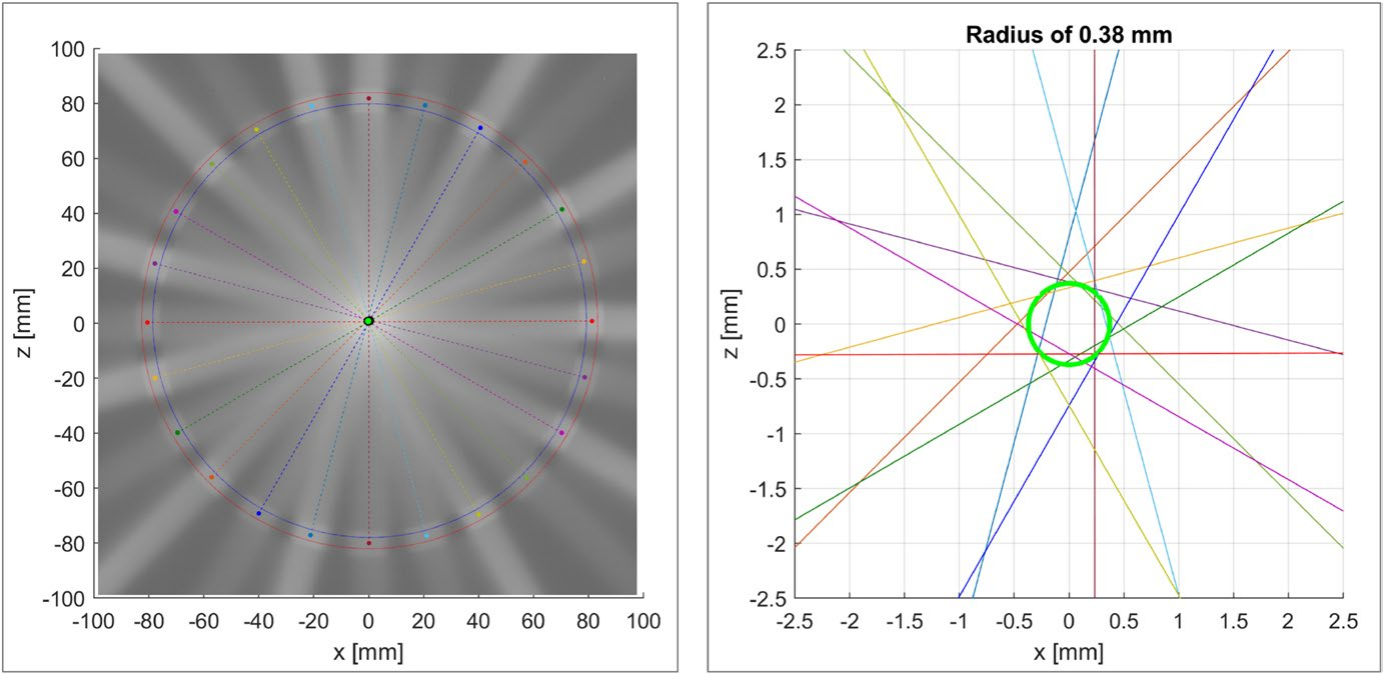
Spoke film acquired with copper rings to assess photon fluence free of Lorentz force interference. The radius of the locus of isocenter was 0.38 mm

The locus of isocenter was measured with a spoke film (see Fig. 6). The radius of the locus was 0.38 mm.

### 3.6 Mechanical and dosimetric alignment of beam limiting devices

**Fig. 7:**
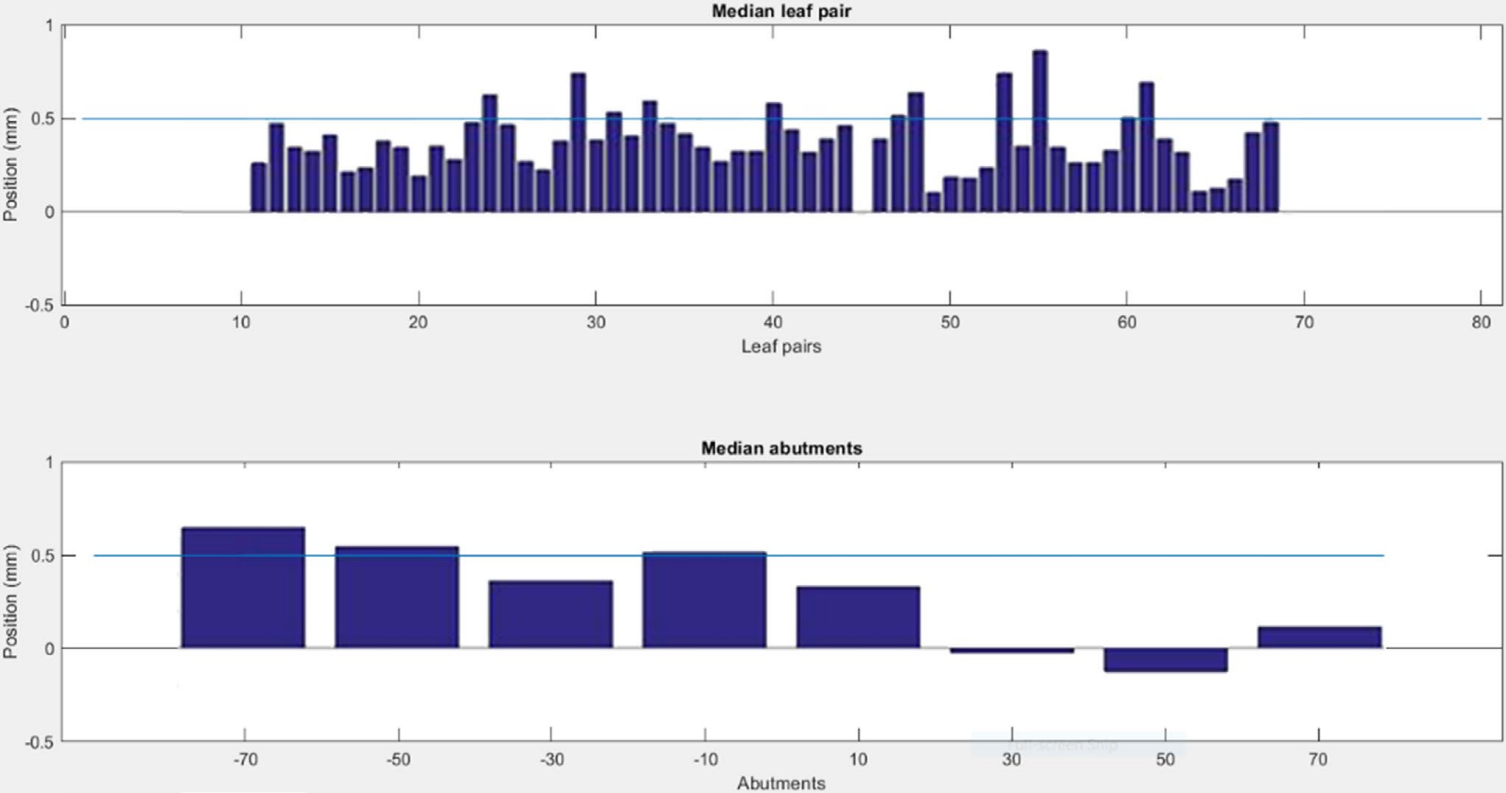
Top — absolute positional error for each leaf pair, averaged over all abutment positions (14 cm region). Bottom — error for each abutment position, averaged over all leaf pairs

The MLC was first assessed at gantry 0°. Over eight different MLC abutment positions, and for 58 leaf pairs, the average absolute positional deviation from the set position was 0.29 mm, with standard deviation 0.41 mm (see Fig. 7). Average relative error between leaf pairs in a stripe was 0.17 mm. Leaf pair 55 had the largest average absolute positional error of 0.8 mm over all positions.

The visual inspection of all 80 leaf pairs imaged from gantry 0° passed.

The x‐jaw positions were measured over the range −200 mm to +150 mm. The maximum deviation from the intended position was 0.9 mm. The average deviation was 0.16 mm. These are well within the traditional specification of 1% (1 mm per 100 mm distance from center).

MLC stripe fields were repeated at gantry 0, 90, 180, and 270 degrees and measured with the MVI, covering 22 cm (30 leaf pairs). The average absolute positional MLC errors, with standard deviations in brackets, were 0.10 (0.23), 0.07 (0.22), −0.08 (0.22), and −0.02 (0.23) mm, respectively.

### 3.7 Table

The table position was checked through a range of motion of 1280 mm. Residual errors in the translational axes were measured. The largest residual error in each (x,y,z) direction was (0.2, 0.4, 0.1) mm. This was within the hospital specification of 1 mm.

Table orthogonality to the gantry plane of rotation was verified by imaging an index bar. The index bar was parallel to the MV image pixel rows to better than 1 pixel in 200 mm (< 0.07 degrees). A set square was used to verify that the index bar was at right angles to the tabletop.

The height of the radiation isocentre above the table top was measured with a spoke film. It was 0.0 ± 0.5 mm different from the nominal value of 140 mm.

### 3.8 Lasers

The Elekta indicative sagittal laser was aligned in a sagittal direction passing through isocenter, suitable to assist with approximate patient setup.

### 3.9 MR to MV alignment

The rotational and translational offsets between the MRI coordinate system and the linac (MV) coordinate system were measured. The rotational differences were (ψ,φ,θ) = (−0.04, +0.03, +0.09) degrees. No correction was applied for the rotational differences. The translational correction was (x,y,z) = (−0.51, −0.52, +0.30) mm.

### 3.10 Dosimetric system

The linac was calibrated consistent with the Netherlands Code of Practice NCS18^35^ with additional correction factors for the influence of the magnetic field.^25^

The linac was reproducible in the short term with standard deviation 0.07%. Linac monitor channel 1 was perfectly 100 MU for each beam (as intended). Monitor channel 2 was always within 0.1 MU (0.1%).

Over a two week period with 10 measurements, the average measured dose was 100.1 ± 0.2% (1σ) of the calibrated value. No trend was apparent. The doses were independent of air pressure.

As part of on‐going QA, measurements were routinely performed with Farmer chamber and MVI over a four month period. From analysis of the standard deviations of the differences of the measurements, it was determined that the standard deviations attributable separately to each of the Farmer chamber in RW3 phantom, MVI and linac were σ = 0.45%, 0.27%, and 0.27%, respectively. The MV imager pixel factor was a valid representation of the phantom‐measured dose and was implemented as a convenient daily dose check.

Linearity with dose was tested over the range 2 – 1000 MU. The difference in cGy/MU for a 5 MU beam was +1.3%, which was within the 2% specification. The difference was less than 0.4% for all longer beams, within the 1% specification. Within each cardinal gantry angle the maximum deviation for 5 MU was less than 1.3%.

Linearity with dose rate was tested for 54, 297, and 418 MU/min. The maximum deviation for different dose rates, and within each cardinal gantry angle was 0.1%.

Inline and crossline IEC symmetry were measured by Elekta with an ICprofiler 2D array on a rotating platform, every 30°. The inline symmetry was in the range 100.4 – 100.9. The crossline symmetry was in the range 100.7 – 102.1. These values were all within the traditional specification of 3% and demonstrated acceptable beam constancy with gantry angle.

The measurements were repeated for the cardinal gantry angles with 5 MU and 100 MU. The differences in symmetry for the low‐MU beams were all less than 0.4%. The maximum in‐field point difference between any beam and the gantry 0° 100 MU beam was 0.8%.

### 3.11 Beam performance during MR imaging

**Fig. 8:**
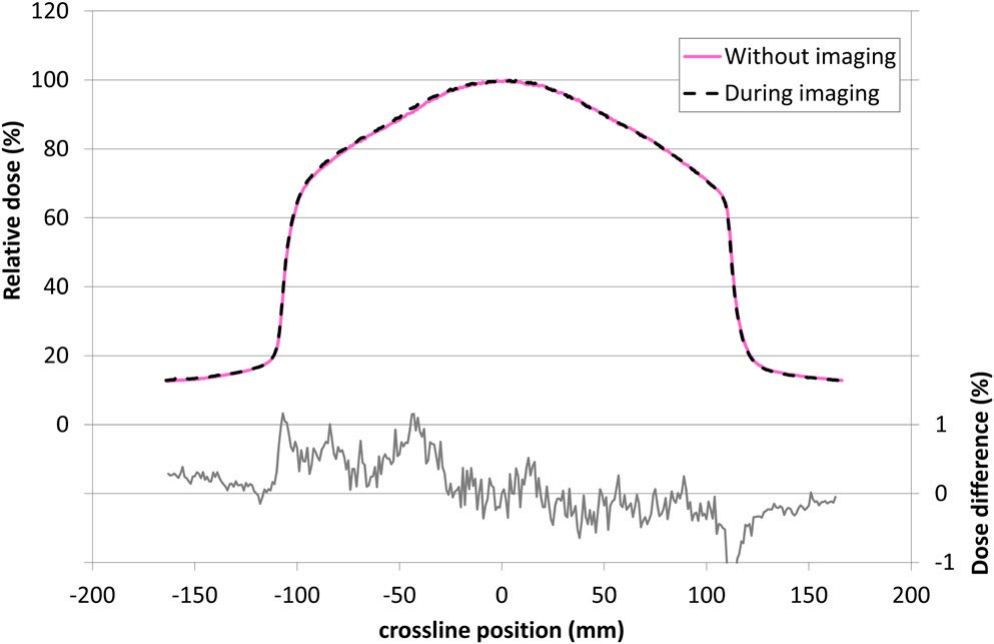
Relative crossline dose profiles and differences, with and without simultaneous MR image acquisition. To within the uncertainties of film dosimetry the profiles were identical

The profiles from the films, with and without MR imaging, were in excellent agreement (Fig. 8). The maximum difference at any point within the beam was 1.1%, consistent with the combined uncertainty of the film measurement and the beam reproducibility.

For the cardinal gantry angles, the maximum difference within the beam, as measured with the MVI, was 0.5%.

Ion chamber measurements were made with and without simultaneous MR imaging. The maximum difference in average readings was −0.13%.

From the ion chamber and film measurements it was concluded that radiation delivery and dose deposition, as expected, were not significantly affected by simultaneous MR imaging.

### 3.12 End to end test

Coordinates and orientations were consistent throughout the Unity system. Gamma test with 5%/2mm parameters and 10% background threshold (SBRT criteria) had 99.8% agreement. Visual inspection of the film exposed within the Alderson phantom showed the dose centered on the target marker. The offset between the co‐registered film and TPS dose distributions was less than 1 mm.

## 4 DISCUSSION

Linac acceptance testing results were acceptable for clinical introduction.

Acceptance tests must be followed by beam data collection, Elekta beam modeling and commissioning.^13^ For the MRI‐linac, beam data collection must include attention to magnetic field‐related effects such as electron streaming^14^ and electron return effect.^12^ Deep PDDs should be acquired with lateral beams, or by stitching together multiple measurements.^13^

Cryostat transmission may vary from one Unity system to the next. It may also vary by 0.9% based on how much helium is in the cryostat. The helium is recycled within the system therefore the helium level does not change appreciably over time, only potentially during specific events (for example magnet ramp‐up). Thus cryostat transmission and helium level must be checked during commissioning.^13^

Table and receiver coil transmission are expected to be the same for all Unity systems. They should be checked as part of the acceptance and commissioning process.^13^

Patient stabilization and support device transmission should be considered as part of the commissioning process. At UMCU transmission through foam supports and vacuum mattress were considered negligible and not included in the dose calculations, consistent with our approach in the regular clinic.

The acceptance testing results were used to establish a baseline for future QA tests.

## 5 CONCLUSION

New tests for the MRI‐linac were developed, implemented and are described here. The MRI‐linac meets safety requirements for patients and operators. The system delivers radiation for SBRT effectively.

## CONFLICT OF INTEREST

David Roberts is an employee of Elekta Limited. UMC Utrecht is a research partner of Elekta AB. The authors have no conflicts of interest to disclose.
